# Prospective Study of the Detection of Bacterial Pathogens in Pediatric Clinical Specimens Using the Melting Temperature Mapping Method

**DOI:** 10.1128/spectrum.00198-22

**Published:** 2022-06-08

**Authors:** Yoji Uejima, Eisuke Suganuma, Takuma Ohnishi, Haruka Takei, Mihoko Furuichi, Satoshi Sato, Yutaka Kawano, Isao Kitajima, Hideki Niimi

**Affiliations:** a Division of Infectious Diseases and Immunology, Saitama Children’s Medical Center, Saitama, Japan; b Department of Clinical Laboratory and Molecular Pathology, Faculty of Medicine, Academic Assembly, University of Toyama, Toyama, Japan; University Paris-Saclay, AP-HP Höpital Antoine Béclère, Service de Microbiologie, Institute for Integrative Biology of the Cell (I2BC), CEA, CNRS; Faculty of Medicine University of Gaziantep; Sakarya University

**Keywords:** 16S RNA, blood culture, bloodstream infections, clinical methods, pediatric infectious disease, rapid tests

## Abstract

The melting temperature (*T_m_*) mapping method is a novel technique that uses seven primer sets without sequencing to detect dominant bacteria. This method can identify pathogenic bacteria in adults within 3 h of blood collection without using conventional culture methods. However, no studies have examined whether pathogenic bacteria can be detected in clinical specimens from pediatric patients with bacterial infections. Here, we designed a new primer set for commercial use, constructed a database with more bacterial species, and examined the agreement rate of bacterial species *in vitro*. Moreover, we investigated whether our system could detect pathogenic bacteria from pediatric patients using the *T_m_* mapping method and compared the detection rates of the *T_m_* mapping and culture methods. A total of 256 pediatric clinical specimens from 156 patients (94 males and 62 females; median age, 2 years [<18 years of age]) were used. The observed concordance rates between the *T_m_* mapping method and the culture method for both positive and negative samples were 76.4% (126/165) in blood samples and 79.1% (72/91) in other clinical specimens. The *T_m_* mapping detection rate was higher than that of culture using both blood and other clinical specimens. In addition, using the *T_m_* mapping method, we identified causative bacteria in pediatric clinical specimens quicker than when using blood cultures. Hence, the *T_m_* mapping method could be a useful adjunct for diagnosing bacterial infections in pediatric patients and may be valuable in antimicrobial stewardship for patients with bacterial infections, especially in culture-negative cases.

**IMPORTANCE** This study provides novel insights regarding the use of the melting temperature (*T_m_*) mapping method to identify the dominant bacteria in samples collected from pediatric patients. We designed a new set of primers for commercial use and developed a database of different bacteria that can be identified using these primers. We show that the *T_m_* mapping method could identify bacteria from blood samples and other clinical specimens. Moreover, we provide evidence that the *T_m_* mapping method has a higher detection rate than that of the culture-based methods and can achieve a relatively high agreement rate. We believe that our study makes a significant contribution to this field because rapid identification of the source of bacterial infections can drastically improve patient outcomes and impede the development of antibiotic-resistant bacteria.

## INTRODUCTION

Traditional culture-based methods of microorganism identification and testing from clinical specimens take several days, during which patients may be receiving ineffective or unnecessary broad-spectrum antibiotics, which may result in poor prognosis (1, 2). Although recent advances in novel diagnostic platforms for bacterial infections, including matrix-assisted laser desorption/ionization time-of-flight mass spectrometry, multiplex PCR assays, 16S ribosomal DNA (rDNA) sequencing, and metagenomic deep sequencing, have improved diagnosis, rapid pathogen identification could promote the early administration of appropriate antimicrobial therapy, thus decelerating the emergence of antimicrobial-resistant organisms, lowering medical expenses, and improving clinical outcomes (3, 4). We previously reported a novel “melting temperature (*T_m_*) mapping method” for rapidly identifying the dominant bacteria in a clinical sample using the 16S rRNA gene (5). This method can identify unknown pathogenic bacteria using 2 mL of whole blood within 3 h after blood collection and without performing a culture test using seven primer sets, without sequencing. This method can also prove the absence of bacteria because it uses a universal bacterial primer. On the other hand, it cannot identify multiple bacteria. However, whether this method can be used for samples from pediatric patients remains unclear.

The circulating blood volume of a child is markedly lower than that of an adult. In newborn babies, especially premature infants, the collection of a large amount of blood may cause a decrease in blood pressure. Thus, the smaller the amount of blood required for the test, the lesser the burden on the circulatory system. The Clinical and Laboratory Standards Institute (CLSI) guideline (6) recommends collecting less than 1% of the total blood volume for blood cultures. In contrast, since numerous pediatric patients have low-level bacteremia (≤10 CFU/mL), recent guidelines from the Infectious Diseases Society of America (IDSA) and the American Society for Microbiology (ASM) recommend that 3 to 4% of the total blood volume be collected for a child with a bodyweight of <12 kg and 1.8 to 2.7% of the total volume for a child with a bodyweight of >12 kg (7). Therefore, the optimal blood volume that should be collected from children for culture studies is not clearly defined. Similarly, the number of clinical specimens required for bacterial identification using the *T_m_* mapping method is not well defined, and there are no comprehensive reports on its usefulness. Hence, in this study, we prospectively evaluated the efficacy of the *T_m_* mapping method in pediatric patients with bacterial infections.

## RESULTS

The sensitivity test results are listed in Table 1. The difference value (*D*) for 0 < *D* ≤ 0.26 was a perfect match (37/37 = 100%), so was for 0.26 < *D* ≤ 0.53 (34/35 = 97%), with a 1/35 = 2.9% mismatch. For 0.53 < *D*, there was a perfect match (2/4 = 50%), a genus match (1/4 = 25%), and a mismatch (1/4 = 25%). For 0.53 < *D*, complete agreement was 50% (2/4), genus agreement was 25% (1/4), and 25% (1/4) were discrepancies. The two samples in which the bacterial counts could not be detected, had low bacterial counts. Therefore, the cutoff value of *D* for identifying clinical samples in this study was set to 0 < *D* < 0.53.

**TABLE 1:** Relationship between D and matches at bacterial count above the minimum detection sensitivity using diluted bacterial samples

Difference value (*D*)	No. of samples	No. of matches	No. of broad matches	No. of mismatches
0 < *D* ≤ 0.26	37	0	0	0
0.26 < *D* ≤ 0.53	35	34	1^*a*^	0
0.53 < *D*	4	2	1	1

a That is, the number of matches at the genus level.

A total of 256 specimens from 156 patients (94 males and 62 females) with a median age of 2 years (range, 0 to 17 years) were collected for this study; nine samples from six patients were discarded from the analysis process due to contamination (eight blood samples and one urine sample; see Table S1 in the supplemental material). In addition to blood samples (*n* = 165), cerebrospinal fluid (CSF; *n* = 41), abscess (*n* = 16), synovial fluid (*n* = 8), urine (*n* = 7), ascites (*n* = 7), and other specimens (*n* = 12) were collected (Table 2). Of the 256 samples, 42 samples (blood, 18; other specimens, 24) from 34 patients were culture positive. The positive results were obtained using the *T_m_* mapping method for 97 samples from 70 patients. The time interval of blood samples between the standard culture and the *T_m_* mapping methods was at a median of 0 h (interquartile range [IQR] = 0 to 0). Among the 165 blood samples, 18 (10.9%) tested positive when the culture method was used, and 53 (32.1%) tested positive when the *T_m_* mapping method was used (Table 3). Among the 91 other clinical specimens from conventionally sterile body sites, 24 (26.4%) tested positive using the culture method, and 43 (47.3%) tested positive using the *T_m_* mapping method (Table 3). Thus, the detection rate of the *T_m_* mapping method was higher than that of the culture method (*P < *0.01). The overall time from filling the automated blood culture device to reporting the results was as follows. The median time to report for blood samples was 6.39 days (IQR = 6.15 to 7.01), and the median time to report for nonblood samples was 3.07 days (IQR = 1.95 to 4.40). On the other hand, the average time from DNA extraction to reporting the results of the *T_m_* mapping method was 3.6 h (range, 2.22 to 3.37).

**TABLE 2:** Clinical characteristics of patients in this study^*a*^

Parameter	No. of patients (*n* = 156)
Median age in yrs (range)	2 (0–17)
No. male	94
Clinical specimens (*n* = 256)	
Blood	165
Specimens other than blood	91
Cerebrospinal fluid	41
Abscess	16
Synovial fluid	8
Urine	7
Ascites	7
Bone tissue	4
Pericardial effusion	4
Pleural effusion	3
Peritoneal dialysis fluid	1

a Contaminated samples (eight blood, one urine) from six patients were excluded from the analysis.

**TABLE 3:** Comparison of pathogenic organism detection capabilities of *T_m_* mapping and conventional culture methods: results from blood samples and from clinical specimens other than blood

Bacterial isolates	*T_m_* mapping method (no. of isolates)			
	Detection	+	−	Total
Conventional culture method (blood samples)^*a*^	+	16* (I = 16)	2†	18
	−	37† (I = 21, NS = 16)	110*	147
	Total	53 (I = 37, NS = 16)	112	165
				
Conventional culture method (other clinical specimens)^*b*^	+	24* (I = 19, NS = 5)	0†	24
	−	19† (I = 11, NS = 8)	48*	67
	Total	43 (I = 30, NS = 13)	48	91

a Fisher exact test *P *<* *0.01; *T_m_*, melting temperature. *, *T_m_* mapping identifications matched the culture results; †, *T_m_* mapping identifications did not match the culture results. I, identified by the *T_m_* mapping method (difference value ≤ 0.53); NS, bacteria were detected, but not suitable for identification by the *T_m_* mapping method (difference value > 0.53).

b McNemar's test *P *<* *0.01.

### Congruence of the culture and *T_m_* mapping method results.

A total of 198 sample results were congruent positive or negative, across the two methods. A total of 40 specimens (16 + 24) showed concordant positive results. Of the 35 specimens identified as positive using the *T_m_* mapping method (*D* ≤ 0.53), 33 specimens were identified and matched, while two samples showed discordance following species identification. The organisms identified were Staphylococcus aureus (*n* = 12), Streptococcus pyogenes (*n* = 4), Klebsiella oxytoca (*n* = 3), Staphylococcus epidermidis (*n* = 3), Enterococcus faecalis (*n* = 2), Escherichia coli (*n* = 2), Streptococcus intermedius (*n* = 2), Enterobacter aerogenes (*n* = 1), Enterococcus faecium (*n* = 1), Klebsiella pneumoniae (*n* = 1), Pseudomonas aeruginosa (*n* = 1), and Streptococcus gallolyticus subsp. *pasteurianus* (*n* = 1). A total of 158 specimens from 108 patients showed negative results according to both methods. Thus, the overall agreement between the *T_m_* mapping method and the culture method for positive and negative samples was 77.3% [Table 3: */total = 198/256]. Including the contamination results, 3.3% of samples were determined to be false positives using the *T_m_* mapping method [contamination/(total + contamination) = 9/265], while 0.75% of samples were false negatives [2/(total + contamination) = 2/265].

### *T_m_* mapping method-positive, culture method-negative specimens.

Of the 56 specimens [Table 3: (37 + 19)] in which bacterial species were identified using the *T_m_* mapping method (*D* ≤ 0.53), 32 specimens from 26 patients were culture negative (Table 4). These samples included 21 blood specimens and four abscesses, two urine, two CSF samples, two ascites samples, and one synovial fluid sample. A “true pathogen” was detected in 13 specimens from 11 patients. These findings were supported by detecting the same pathogen from the same infectious site in eight specimens, and the detection of the same pathogen in other sites in five specimens. Thirty-two specimens from 38 patients had received adequate antimicrobial treatment before collecting clinical specimens.

**TABLE 4:** Pathogen types

Pathogen category and reference no.	Specimen type	Clinical diagnosis	Organism detected by *T_m_* mapping	Antibiotic(s) administered before sample collection^*a*^	Specimens that tested positive by another method (time)^*b*^
“True” pathogens					
22	Abscess	Sepsis, infected simple renal cyst	Streptococcus pneumoniae	PIPC/TAZ	Same pathogen in blood by culture (d23–)
49	Urine	UTI	Escherichia coli	CTX	Same pathogen in urine by culture (d1–)
65	Blood	Sepsis	Enterococcus faecalis	ABPC, GEN	Same pathogen in blood by culture (d13–)
82	Blood	Sepsis	Streptococcus pyogenes	ABPC, CTX	Same pathogen in blood by culture (d1–)
84	Blood	Sepsis	Streptococcus pyogenes	ABPC, ABK, CLDM	Same pathogen in blood by culture (d8–)
102	Ascites	Peritonitis	Pseudomonas aeruginosa	CFPM, VCM	Same pathogen in drainage tube by culture
120	Blood	Sepsis, early-onset GBS infection	Streptococcus agalactiae	PIPC (maternal antibiotic exposure)	same pathogen in skin and stool by culture
137	CSF	Meningitis	Staphylococcus capitis subsp. *ureolyticus*	VCM	same pathogen in CSF by culture (d7–)
138	CSF	Meningitis	Staphylococcus capitis subsp. *ureolyticus*	VCM	same pathogen in CSF by culture (d17–)
150	Blood	Sepsis, septic arthritis	Staphylococcus aureus	None	Same pathogen in hip joint by culture (d1+)
175	Blood	Meningitis	Streptococcus agalactiae	ABPC, CTX	Same pathogen in CSF by culture (d3–)
186	Blood	Meningitis, sepsis	Streptococcus agalactiae	ABPC, CTX	Same pathogen in blood by culture (d1–)
204	Blood	Sepsis	Streptococcus agalactiae	ABPC	Same pathogen in skin, gastric juice, and pharyngeal mucus by culture
					
“Possible” pathogens					
9	Blood	Sepsis	Staphylococcus warneri	SBT/ABPC	None
70	Blood	Sepsis	Cutibacterium acnes	MEPM	None
75	Abscess	Pyriform sinus fistula- associated infections	Bacillus cereus	ABPC, CTX	None
116	Blood	Bacteremia, CVID	Cutibacterium acnes	CTRX	None
118	Blood	Bacteremia, CVID	Cutibacterium acnes	CTRX	None
115	Blood	Bacteremia, CVID	Cutibacterium acnes	CTRX	None
134	Blood	Sepsis, necrotizing fasciitis	Corynebacterium xerosis	ABPC, CTX, TEIC	None
135	Blood	Necrotizing enterocolitis	Clostridium butyricum	VCM, MEPM	None
162	Blood	Sepsis	Staphylococcus haemolyticus	ABPC, AMK	None
164	Synovial fluid	Septic arthritis	Streptococcus pneumoniae	CEZ	None
168	Ascites	Perforated appendicitis	Acinetobacter baumannii	PAPM/BP	None
177	Urine	UTI	Finegoldia magna	ABPC, CTX	None
197	Blood	MAS, sepsis	Fusobacterium nucleatum	ABPC, CTX	Same pathogen in blood by 16S rDNA sequence
217	Abscess	Subdural empyema	Streptococcus intermedius	CTRX, VCM	Same pathogen in abscess by 16S rDNA sequence
230	Blood	Bacteremia	Cutibacterium acnes	SBT/ABPC	None
254	Abscess	Lymphadenitis	Staphylococcus aureus	SBT/ABPC	None
“Indeterminate” pathogens					
103	Blood	Cellulitis	Clostridium perfringens	CEZ	*Kocuria spp.* in blood by 16S rDNA
256	Blood	CRMO	Cutibacterium acnes	LVFX, CAM	None
257	Blood	CRMO	Cutibacterium acnes	None	None

a AMK, amikacin; ABPC, ampicillin; ABK, arbekacin; CRBSI, catheter-related bloodstream infection; CEZ, cefazolin; CTX, cefotaxime; CTRX, ceftriaxone; CSF, cerebrospinal fluid; CRMO, chronic recurrent multifocal osteomyelitis; CVID, common variable immunodeficiency; CAM, clarithromycin; CLDM, clindamycin; GEN, gentamicin; GBS, group B Streptococcus; LVFX, levofloxacin; MAS, Meconium aspiration syndrome; MEPM, meropenem; PAPM/BP, panipenem/betamipron; PIPC/TAZ, piperacillin-tazobactam; SBT/ABPC, sulbactam/ampicillin; TEIC, teicoplanin; UTI, urinary tract infection; VCM, vancomycin.

b Time point of pathogen detection. d, days; –, pathogen detection before sampling; +, pathogen detection after sampling.

### *T_m_* mapping-negative, culture-positive results.

Two blood samples from two patients tested positive for pathogens when the culture method was used but were negative when the *T_m_* mapping method was used. One sample contained Streptococcus pneumoniae, while the other contained Salmonella enteritidis.

### Discordance in species identification.

In two samples from two patients, different organisms were identified using the culture and *T_m_* mapping methods; we found consistency in one of these samples at the genus level (Staphylococcus aureus versus Staphylococcus cohnii). In the other sample with discordant species identification, the culture method identified Staphylococcus aureus from multiple sample specimens collected from the same infectious site. In contrast, the *T_m_* mapping method identified the pathogen to be Prevotella bivia.

## DISCUSSION

To the best of our knowledge, this is the first study to investigate the detection rate of bacterial pathogens in clinical specimens collected from children using the *T_m_* mapping method. This prospective study revealed that in pediatric patients, the *T_m_* mapping method is associated with a higher pathogen detection rate than the classical culture method. Moreover, the higher detection rate was not restricted to blood samples. Comparing our findings to those of a previous study (5), the accuracy of the *T_m_* mapping method using whole-blood samples versus the culture method was slightly lower (76.4% versus 85.5%). Two main reasons can justify this discrepancy. First, since the rate of positive results using the culture method was lower in this study compared to that of the previous study (5) (10.9% versus 22.5%), it is presumed that there are differences in the patient background information, such as differences in the collection timing and the proportion of patients administered antibiotics. Second, in this study, the primers and databases used for the *T_m_* mapping method were those developed for commercial use. Therefore, the detection sensitivity might be lower than that in previously published data.

We used specific criteria to classify the detected pathogen as a true, possible, contamination, or indeterminate pathogen for culture-negative specimens that tested positive using the *T_m_* mapping method. Of the 41 specimens detected using the *T_m_* mapping method, only 13 were considered to contain true pathogens, while 16 of them were categorized as samples harboring possible pathogens, nine were categorized as contamination pathogens, and three were considered indeterminate. Upon further investigation of the culture method results from the same infectious site, eight samples from seven patients were considered true pathogens, whereas five specimens from five patients were verified using the culture method results using samples from other sites. We also identified 16 specimens from 14 patients as possible pathogens, of which the results associated with two specimens were confirmed by identifying the same pathogen at the same infectious site using 16S rDNA sequencing, and the results of 14 specimens were supported by case reports of infections due to Corynebacterium xerosis (8–10), Cutibacterium acnes (Propionibacterium acnes) (11–13), Staphylococcus haemolyticus (14–16), and Staphylococcus warneri (17–19) in patients with sepsis or bacteremia. In addition, fastidious or noncultivable organisms, namely, Clostridium butyricum (20, 21), Finegoldia magna (22, 23), and Fusobacterium nucleatum (24, 25), were detected in necrotizing enterocolitis, urinary tract infection, and sepsis, respectively. Moreover, we identified Streptococcus pneumoniae (26, 27) from septic arthritis, Acinetobacter baumannii (28, 29) in ascites from a patient with perforated appendicitis, Streptococcus intermedius (30, 31) from subdural empyema, and Staphylococcus aureus (32–34) from lymphadenitis. All specimens with true and possible pathogens (except one blood specimen from septic arthritis) were collected following antibiotic treatment. This could explain the negative culture results in these specimens. The initiation of empirical antibiotic pretreatment among patients with sepsis significantly reduces the likelihood of obtaining positive blood cultures drawn shortly after treatment initiation (35). Culture tests do not detect bacteria that have died due to leukocyte phagocytosis or antibiotic administration. Conversely, in this test, since a buffy coat containing many white blood cells with phagocytosed bacteria is collected, dead bacteria can also be detected. Opota et al. (36) stated that one of the limitations that must be faced with respect to detecting bacteria by PCR amplification in blood samples is the presence of DNA from dead microorganisms. The *T_m_* mapping method also uses PCR-based amplification of bacterial DNA. It is a testing method that also detects dead bacteria. It is necessary to avoid defining dead bacteria as infectious and subjecting patients to unnecessary antibiotic therapy. However, we reported a case in which nucleic acid of Streptococcus pneumoniae was detected in the cyst of a patient with an active, infected simple renal cyst after antimicrobial therapy, and although culture was negative, antimicrobial de-escalation could be performed based on the results of the *T_m_* mapping method (37). Thus, the *T_m_* mapping method can be very useful as a test for some of the clinical information about bacterial infections, especially in identifying the nucleic acids of dead bacteria in patients who have received prior antimicrobial therapy.

Among the organisms classified as contamination pathogens, *C. acnes* (P. acnes) was the most common, followed by staphylococcal bacteria and Corynebacterium xerosis. More than one blood culture sample must be positive with the same isolate to avoid being considered contaminated with clinical specimens by commensal microorganisms to distinguish contamination from true pathogens among skin and bloodstream infections. However, the PCR-based method exhibits a higher detection rate of *C. acnes* compared to the culture method (38). Thus, since the *T_m_* mapping method is based on PCR with high detection sensitivity, contamination is also likely to occur regularly. Thorough sterilization at the time of sample collection, DNA extraction, and mechanization of the *T_m_* mapping method are required to prevent the contamination of commensal bacteria from the environment.

We could not determine the association between the detected pathogen and the disease in the indeterminate cases. For instance, although *C. acnes* has been detected in patients with CRMO, it remains unclear whether *C. acnes* is a pathogenetic organism (39). Although *C. acnes* could produce acute infections (40), the patient improved without antibiotics. The *T_m_* mapping method also detected Clostridium perfringens, whereas *Kocuria* spp. was detected using the 16S rDNA. Therefore, a mixed infection with no dominant species was identified as the likely cause.

The result may be considered a false-negative for the two culture-positive specimens that tested negative after using the *T_m_* mapping method. This false-negative result could be explained using human serum DNases, which are known to degrade bacterial DNA. Heininger et al. (41) reported that PCR-based detection of E. coli in serum was reduced by 10% after antibiotic treatment. Residual bacterial DNA may be detected using the PCR method after antibiotic treatment, even at low levels of bacterial DNA. The bacterial DNA extracted from 2 mL of blood is ultimately concentrated into 50 μL, of which 2 μL is used for the *T_m_* mapping method. Therefore, theoretically, if there is even one bacterial species in the 2-μL sample, it will be amplified and detected. We estimate that small bacterial DNA might not be inserted into the *T_m_* mapping method but only into the culture method.

Using the *T_m_* mapping method, dominant bacteria from clinical specimens were amplified because nucleic acids are amplified by PCR. Moreover, the seven *T_m_* values overlap when specimens contain similar amounts of multiple bacteria, making it difficult to identify the causative organism. Therefore, the *T_m_* mapping method is mainly suitable for identifying a single bacterium in a sterile sample. When identifying multiple infection-inducing bacteria, such as sputum and perianal abscess, only the dominant bacteria can or cannot be identified depending on the ratio of the bacterial mass, which was discovered to be the case for some of the indeterminate cases. In addition, this method can only identify bacteria because PCR is performed using universal bacterial primers. Since *T_m_* mapping can directly identify bacteria from clinical samples without a culture assay, it could be used, particularly in cases where rapid testing is required or the detection of dead bacteria following antibiotic treatment is desired. This method could be generalized by simplifying and mechanizing it to ensure that contamination does not occur in the inspection process.

Our study has several limitations. The present study has confounding factors and biases, such as those in the ages of pediatric patients from whom clinical specimens were collected and the small number of clinical specimens obtained. Moreover, most samples for the *T_m_* mapping method were collected after antibacterial drug administration for the blood sample analysis. Hence, the detection rate of bacteria using *T_m_* mapping could be underestimated because it was compared to that of the culture method, which tested samples collected before the antibiotic was administered. Since there is no data on the amount of blood filled in the blood culture bottles, the possibility cannot be ruled out that the higher the amount of blood, the higher the positive rate of blood culture. In addition, not all 16S rDNA phylogenetic analyses could have been performed on all specimens. As of July 2019, the 162 pathogenic bacterial strains from adult patients with sepsis have been registered in the database of the sepsis-causing bacterium identification system using the *T_m_* mapping method, and each bacterial species includes two to three mutant strains. In addition, this *T_m_* mapping method does not provide information on antibiotic susceptibility tests and colony counts, which culture methods can confirm. Therefore, it is incomplete as a guide to treatment and is not a replacement for a conventional culture test. In the conventional culture method, fungi may be detected in the culture medium, but no fungi are detected in this method. In addition, consideration should be given to how to intervene in treatment in light of the clinical course with respect to detecting dead bacteria to avoid unnecessary antimicrobial exposure. Finally, this study was limited to patients at a single center. However, our hospital is located in the center of the prefecture, and as a tertiary medical institution, healthy and immunocompromised patients with various diseases visit the hospital. Although the false-positive rate was not shown in the previous study performed at a different hospital, the false-negative rate was comparable (0.75% versus 1%).

In conclusion, the *T_m_* mapping method appears to be a useful tool for diagnosing various bacterial infections in children. Although various testing tools have been developed, appropriate treatment strategies must be selected according to the clinical information of the patients and the results of the tests. For example, antibiotics were changed to narrow-spectrum antibacterial drugs, or the treatment was supported after identifying the bacterial species in some cases. Hence, more cases must be studied in detail using this method to identify the optimal treatment method for bacterial infections and the appropriate use of antibiotics.

## MATERIALS AND METHODS

### Sensitivity test.

We performed a sensitivity test for commercial use using newly constructed primer sets and a new database. The procedures were as follows: each bacterium was cultured purely in Luria Bertani (BD Difco, Franklin Lakes, NJ) medium, and the number of microbial cells per milliliter in the culture solution of each sample was measured using a flow cytometer (Beckman Coulter CytoFLEX, catalog no. B53019). Thereafter, the microbial cell density in the culture solution was adjusted by inoculating 2 mL of blood with each bacterial culture solution. The type strain was obtained from JCM (Japan Collection of Microorganisms, RIKEN BioResource Research Center, Tsukuba, Japan). The minimum detectable concentrations for each bacterium were as follows: Escherichia coli (JCM 1649^T^), Enterobacter cloacae (JCM 1232^T^), Enterococcus faecalis (JCM 5803^T^), and Klebsiella pneumoniae (JCM 1662^T^) were identified at 10 to 20 cells/mL, while Pseudomonas aeruginosa (JCM 5962^T^), Staphylococcus aureus (JCM 20624^T^, JCM 2151, JCM 8704, and JCM 16555), and Staphylococcus epidermidis (JCM 2414^T^) were identified at 20 to 40 cells/mL. Thereafter, the bacterial DNA was extracted from the collected bacterial pellets by crushing them with glass beads (high purity PCR template kit, Roche, Mannheim, Germany). Lastly, the bacteria were identified using the *T_m_* mapping method.

### Setting and participants.

This is a prospective single-center study that took place at the Saitama Children’s Medical Center from January 2015 to April 2020. Eligible subjects included patients suspected of bacterial infection less than 18 years of age. One bacterial infection event was clinically determined by the attending physician and the infection consulting team, one case at a time, prospectively depending on the type of infection, including signs and symptoms, laboratory results, and completion of antimicrobial therapy. Blood was collected from subjects and aseptically incubated in BacT/Alert PF plus (up to 4 mL) and BacT/Alert FN plus (up to 10 mL) (bioMérieux, Marcy l’Étoile, France) bottles for culture (pediatric bottles and anaerobic bottles). The attending physician determined the sample volume of blood culture based on clinical settings, including medical condition and weight. The attending physician collected one or two sets of blood cultures based on the patient’s general condition. For *T_m_* mapping,0.5 to 2 mL of whole blood was collected in sterile tubes (Neotube; NIPRO, Osaka, Japan) free of DNA contamination and sent to the laboratory. Other clinical samples were collected in sterile screw-cap tubes for both the traditional culturing and *T_m_* mapping methods. Since there was a delay between sample collection for blood culture and *T_m_* mapping, we limited our comparisons to samples collected from the same patient within 24 h. Blood specimens for *T_m_* mapping were collected after collecting specimens for culturing. Clinical specimens, such as CSF, ascites, and pus, were divided into two sterile screw-cap tubes for culturing and *T_m_* mapping. Clinical specimens that did not meet the criteria presented above (e.g., if the blood volume was <0.5 mL or the time between the culture and *T_m_* mapping specimen collection exceeded 24 h) were not considered comparable between methods; therefore, these specimens were excluded from the analysis. If one or both bottles of a blood culture set was positive, it was counted only once as a positive blood culture set. The time for the extraction of pathogenic microorganisms is shorter if several samples collected simultaneously yield positive results.

### Culture.

After collection, the specimens were sent to a laboratory, and the blood was cultured using a BacT/Alert three-dimensional (bioMérieux) automated blood culture system. Gram staining was performed directly from the blood culture bottle in the case of blood culture. In addition, aliquots of in-bottle fluid were aseptically removed from positive bottles where the bacteria had developed using standard methods and inoculated onto sheep blood agar, chocolate agar, and Bromothymol Blue lactose agar media. If anaerobic bacteria were presumed by Gram staining of the culture medium, the laboratory technician added isolation media for anaerobic bacteria. Blood culture bottles that did not test positive in the system for 6 days were defined as negative. For culture methods for clinical specimens other than blood, general bacterial isolation and culture intensification were performed by inoculating directly into the medium with sterile platinum ears. In addition to the common basic media, since the species of bacteria detected differed depending on the specimen, additional media, including selective enrichment broths, were selected by the clinical technologist based on smear results, clinical information, etc. Bacterial isolates were subjected to biochemical tests for identification and classification (7). The isolates were identified at the hospital laboratory using the MicroScan WalkAway 40 SI between 2014 and 2016 and the MicroScan WalkAway 96 Plus between 2017 and 2020.

### DNA isolation.

Bacterial DNA was isolated from the clinical specimens using DNA extraction kits (a high pure PCR template kit [Roche, Mannheim, Germany] from January 2015 to November 2018 and a DNA Extraction kit [Mitsui Chemicals, Tokyo, Japan] from December 2018 to April 2020) according to the manufacturer’s instructions, and eluates were stored at −20°C. DNA isolation was performed in a laminar flow biosafety cabinet decontaminated daily by UV radiation, and strict separation from the PCR was maintained to prevent DNA contamination.

### *T_m_* mapping method.

The procedure of the *T_m_* mapping method has been described in detail elsewhere (5). Briefly, the first PCR was performed using the eukaryote-produced thermostable DNA (Taq) polymerase (Mitsui Chemicals, Tokyo, Japan), which has no contamination of bacterial DNA, and one universal bacterial primer (the bacterial conserved region of the 16S rRNA gene, which is a primer for PCR detection of all bacteria). A negative-control sample consisting of sterile water (nontemplate sample) and a positive-control sample (e.g., E. coli ATCC 25922) were included in each experiment. The used amplification protocol was as follows: 95°C for 5 min, followed by 30 cycles of 94°C for 10 s, 65°C for 20 s, 72°C for 30 s, and 85°C for 2 s. Subsequently, the PCR product was diluted 500-fold using molecular-grade distilled water (water deionized and sterilized for molecular biology, Nacalai Tesque, Inc., Japan) and used as a template for the second (nested) PCR procedure. Subsequently, seven universal bacterial primers targeting conserved regions of the 16S rRNA genes were used in the second PCR. The primers developed for commercial use were as follows: region 1 primers (forward, 5′-GCAGGCTTAACACATGCAAGTCG-3′; reverse, 5′-CGTAGGAGTCTGGACCGT-3′), region 2 primers (forward, 5′-GTCCAGACTCCTACGGGAG-3′; reverse, 5′-CCTACGTATTACCGCGG-3′), region 3 primers (forward, 5′-AGCAGCCGCGGTAATA-3′; reverse, 5′-GGACTACCAGGGTATCTAATCCT-3′), region 4 primers (forward, 5′-AACAGGATTAGATACCCTGGTAG-3′; reverse, 5′-AATTAAACCACATGCTCCACC-3′), region 5 primers (forward, 5′-TGGTTTAATTCGATGCAACGC-3′; reverse, 5′-GAGCTGACGACAGCCAT-3′), region 6 primers (forward, 5′-GTTAAGTCCCGCAACGAG-3′; reverse, 5′-CCATTGTAGCACGTGTGTAG-3′), and region 7 primers (forward, 5′-GGCTACACACGTGCTACAATGG-3′; reverse, 5′-AGACCCGGGAACGTATTC-3′). The amplification protocol used during the second step was similar to the previous one: 95°C for 5 min, followed by 35 cycles of 94°C for 10 s, 60°C for 20 s, 72°C for 30 s, and 85°C for 2 s. For the *T_m_* analysis, the resulting amplicons were first heated at 95°C for 10 s and then cooled at 72°C for 90 s. Afterward, the temperature was gradually increased from 72 to 95°C, at a rate of 0.5°C/step. The data profile was analyzed using a Rotor-Gene Q (Qiagen, Germany). Subsequently, we measured the *T_m_* values of the seven PCR amplicons and mapped them onto two dimensions. We identified the bacteria by comparing them with the bacterial species registered in the database (Rapid Diagnostic System for Bacterial Identification; Mitsui Chemicals, Tokyo, Japan). The accuracy of the identification was evaluated using *D*.

### Interpretation criteria for discrepancies in results.

Two or more physicians, including the treating physician, evaluated several criteria while considering the clinical status of the patient. A nucleic acid of the pathogen detected using *T_m_* mapping was defined as a “true pathogen” in cases where this pathogen was cultured from additional specimens collected from the same infectious site during the same infectious episode and/or the species was specific to the type of infection seen in the patient. A nucleic acid of the pathogen was termed a “possible pathogen” if it had been previously reported as a causative agent of infection and was detected by only one method. Alternatively, isolation of a nucleic acid of a common contaminant from the clinical specimen by the *T_m_* mapping method without a positive result of blood culture; judged by the attending physician and Infectious Diseases Consultant to be a contaminant, and no treatment initiated, was termed “contamination pathogens.” The microorganisms with positive PCR results that met none of the other criteria were designated “indeterminate.” A pathogen identified only by culturing was regarded as a “true pathogen,” since culturing is considered the gold standard for identifying microorganisms.

### Statistical analyses.

We compared the *T_m_* mapping and culturing methods using Fisher exact test to detect pathogens from blood samples and the McNemar's test for other clinical specimens. Statistical analyses were performed using the EZR v1.40 software. Differences were considered statistically significant at two-tailed *P* values of <0.05.

### Data availability.

The data sets generated during and/or analyzed during the present study are available from the figshare repository (https://doi.org/10.6084/m9.figshare.19727236).

## Supplementary Material

Reviewer comments
