# Supplementary material for: Prospective Study of the Detection of Bacterial Pathogens in Pediatric Clinical Specimens Using the Melting Temperature Mapping Method
Source: Microbiol Spectr. 2022 Jun 8;10(3):e00198-22. doi: 10.1128/spectrum.00198-22 (PMC9241829; doi:10.1128/spectrum.00198-22)
Supplement: Supplemental file 1 — Table S1. Download spectrum.00198-22-s0001.pdf, PDF file, 0.7 MB [file spectrum.00198-22-s0001.pdf]

Supplementary Table 1. Details of types of clinical specimens, culture results and Tm mapping results. All specimens analyzed in this study are described in this table, with specimens in which the results are congruent between the culture and Tm mapping methods (whether positive or negative) being highlighted in pink (coloration of the sample number cell). Culture-negative specimens that tested positive with Tm mapping identification (Difference value ≤ 0.53) were categorized as "true pathogens" and are highlighted in yellow, while those categorized as "possible pathogens" are highlighted in green. Specimens categorized as "contamination pathogens" are highlighted in gray, while those categorized as "indeterminate pathogens" are highlighted in blue. Culture-negative specimens that tested positive with Tm mapping but not suitable for Tm mapping identification (Difference Value >0.53) are non-hilighted. Finally, culture-positive specimens that tested negative with Tm mapping are highlighted in purple.

Sample Number	Specimen type	Clinical diagnosis	Culture results	Tm mapping method results
1	Blood	URI	-	-
2	CSF	URI	-	-
3	Blood	Drug eruptions	-	*Cutibacterium acnes*
4	Blood	Sepsis, Septic arthritis	*Staphylococcus aureus*	*Staphylococcus aureus*
5	Blood	Sepsis, Septic arthritis	*Staphylococcus aureus*	*Prevotella bivia*
6	Synovial fluid	Sepsis, Septic arthritis	*Staphylococcus aureus*	*Staphylococcus aureus*
7	Blood	AML, FN	-	-
8	Pleural effusion	Pleural empyema	*Staphylococcus aureus*	*Staphylococcus aureus*
9	Blood	Sepsis	-	*Staphylococcus warneri*
10	Urine	Sepsis	-	-
11	Blood	Postoperative inflammation	-	-
12	Bone tissue	CRMO	-	-
13	Blood	AML, FN	-	-
14	Blood	HPS	-	-
15	Blood	sJIA	-	-
16	Blood	Lymphadenitis	-	+
17	Blood	URI	-	-
18	Blood	Congenital syphilis	-	+
19	Blood	Neonatal pulmonary hemorrhage	-	-
20	Blood	Neonatal pulmonary hemorrhage	-	-

21	Pleural effusion	Sepsis, Infected simple renal cyst	-	-
22	Abscess	Sepsis, Infected simple renal cyst	-	*Streptococcus pneumoniae*
23	Blood	UTI	-	-
24	Urine	UTI	*Enterobacter aerogenes*	*Enterobacter aerogenes*
25	Blood	UTI	-	-
26	Urine	UTI	-	-
27	Blood	UTI	-	-
28	Abscess	Deep neck abscess	*Streptococcus pyogenes*	*Streptococcus pyogenes*
29	Blood	Kawasaki disease	-	-
30	Blood	Meningitis	-	-
31	CSF	Meningitis	-	+
32	Blood	Meningitis	-	-
33	CSF	Meningitis	-	+
34	CSF	Meningitis	-	+
35	CSF	Meningitis	-	+
36	CSF	Meningitis	-	-
37	CSF	Meningitis	-	-
38	CSF	Meningitis	-	-
39	CSF	Meningitis	-	-
40	CSF	Meningitis	-	-
41	Blood	Neonatal myocardial infarction	-	-
42	Blood	Liver abscess	*Klebsiella pneumoniae*	*Klebsiella pneumoniae*
43	CSF	Meningitis	-	-
44	CSF	Meningitis	*Streptococcus intermedius*	*Streptococcus intermedius*
45	Blood	Meningitis	-	-
46	Blood	UTI	-	*Staphylococcu s aureus*
47	Urine	UTI	*Escherichia coli*	*Escherichia coli*
48	Blood	UTI	-	-
49	Urine	UTI	-	*Escherichia coli*
50	Blood	UTI	-	-
51	Urine	UTI	-	-
52	Blood	Kawasaki disease	-	-
53	Blood	Burn, Sepsis	-	-
54	Blood	Methylmalonic acidemia	-	-

55	CSF	Methylmalonic acidemia	-	-
56	Blood	Neonatal fever	-	-
57	Blood	HHV-6 encephalitis	-	*Cutibacterium acnes*
58	Blood	Neonatal fever	-	-
59	Blood	Meconium aspiration syndrome	-	-
60	Blood	Sepsis	*Streptococcus gallolyticus subsp. pasteurianus*	*Streptococcus gallolyticus subsp. pasteurianus*
61	Blood	Neonatal pulmonary hemorrhage	-	-
62	Blood	Invasive fungal disease	-	*Cutibacterium acnes*
63	Blood	Sepsis, UTI	*Enterococcus faecalis*	*Enterococcus faecalis*
64	Blood	Sepsis, UTI	-	+
65	Blood	Sepsis, UTI	-	*Enterococcus faecalis*
66	Blood	Sepsis, UTI	-	-
67	Blood	Pulmonary abscess	-	-
68	Blood	Pneumonitis	*Streptococcus pneumoniae*	-
69	Blood	SLE	-	-
70	Blood	Sepsis	-	*Cutibacterium acnes*
71	Blood	Sepsis	*Staphylococcus epidermidis*	*Staphylococcu s epidermidis*
72	Blood	ALL、FN	-	-
73	Blood	Deep neck abscess	-	-
74	Abscess	Deep neck abscess	*Streptococcus pyogenes*	*Streptococcus pyogenes*
75	Abscess	Pyriform sinus fistula associated infections	-	*Bacillus cereus*
76	Blood	Norovirus infection	-	*Cutibacterium acnes*
77	Pleural effusion	Pulmonary empyema	-	+
78	Blood	Necrotizing enterocolitis	-	-
79	Blood	Necrotizing enterocolitis	-	-
80	Blood	Intracranial hemorrhage	-	-

81	Blood	Sepsis, Infectious endocarditis	*Streptococcus pyogenes*	*Streptococcus pyogenes*
82	Blood	Sepsis, Infectious endocarditis	-	*Streptococcus pyogenes*
83	Blood	Sepsis, Infectious endocarditis	-	*Corynebacterium xerosis*
84	Blood	Sepsis, Infectious endocarditis	-	*Streptococcus pyogenes*
85	Blood	Sepsis, Infectious endocarditis	-	-
86	Blood	Bacterial myositis	-	-
87	Blood	Sepsis, Fulminant hepatitis	*Staphylococcus aureus*	*Staphylococcus aureus*
88	CSF	Meningitis	-	-
89	Blood	Meningitis	-	-
90	Blood	AML, Liver abscess	-	-
91	Blood	Pulmonary abscess	-	-
92	Blood	CRMO	-	-
93	Blood	CRMO	-	-
94	Urine	Sepsis	-	*Staphylococcus aureus*
95	CSF	Intracranial hemorrhage	-	-
96	Ascites	Sepsis, Peritonitis	*Enterobacter asburiae*	+
97	Ascites	Sepsis, Peritonitis	*Citrobacter freundii*	+
98	Blood	Castleman disease	-	-
99	Blood	Cholangitis	-	+
100	Blood	Cholangitis	-	*Staphylococcus capitis subsp. ureolyticus*
101	Blood	Peritonitis	-	-
102	Ascites	Peritonitis	-	*Pseudomonas aeruginosa*
103	Blood	Cellulitis	-	*Clostridium perfringens*
104	CSF	Cellulitis	-	-
105	Blood	Cellulitis	-	-
106	CSF	Cellulitis	-	-
107	Blood	Ventriculoperitoneal infection	-	-
108	CSF	Ventriculoperitoneal infection	*Staphylococcus aureus*	*Staphylococcus aureus*
109	CSF	Ventriculoperitoneal infection	*Staphylococcus aureus*	*Staphylococcus aureus*

110	CSF	Ventriculoperitoneal infection	-	-
111	CSF	Ventriculoperitoneal infection	-	-
112	Blood	ALL, Liver abscess	-	-
113	Blood	Sepsis, CHD	*Klebsiella oxytoca*	*Klebsiella oxytoca*
114	Blood	Sepsis, Invasive meningococcal disease	-	+
115	Blood	Bacteremia, CVID	-	*Cutibacterium acnes*
116	Blood	Bacteremia, CVID	-	*Cutibacterium acnes*
117	CSF	Bacteremia, CVID	-	-
118	Blood	Bacteremia, CVID	-	*Cutibacterium acnes*
119	CSF	Bacteremia, CVID	-	-
120	Blood	Sepsis, Early-onset GBS infection	-	*Streptococcus agalactiae*
121	CSF	Sepsis, Early-onset GBS infection	-	-
122	Synovial fluid	Septic arthritis	-	-
123	Blood	Necrotizing enterocolitis	-	+
124	Blood	Necrotizing enterocolitis	-	+
125	Blood	Necrotizing enterocolitis	-	-
126	Blood	Necrotizing enterocolitis	-	-
127	Blood	Adenovirus infection	-	-
128	Blood	Sepsis	*Salmonella enteritidis*	-
129	CSF	Meningitis	*Staphylococcus epidermidis*	*Staphylococcus epidermidis*
130	CSF	Meningitis	*Staphylococcus epidermidis*	*Staphylococcus epidermidis*
131	CSF	Meningitis	-	-
132	Blood	Sepsis	*Klebsiella oxytoca*	*Klebsiella oxytoca*
133	Blood	Sepsis	*Klebsiella oxytoca*	*Klebsiella oxytoca*

134	Blood	Sepsis, Necrotizing fasciitis	-	*Corynebacterium xerosis*
135	Blood	Necrotizing enterocolitis	-	*Clostridium butyricum*
136	Blood	Shock, Esophageal perforation	-	-
137	CSF	Meningitis	-	*Staphylococcus capitis subsp. ureolyticus*
138	CSF	Meningitis	-	*Staphylococcus capitis subsp. ureolyticus*
139	CSF	Meningitis	-	-
140	Blood	Acute encephalitis	-	+
141	Blood	Acute encephalitis	-	-
142	Bone tissue	Bone fracture	-	-
143	Blood	MCTD, Macrophage activation syndrome	-	-
144	Blood	Acute myocarditis	-	-
145	Blood	Kawasaki disease	-	-
146	Blood	pJIA	-	-
147	Blood	Sepsis	-	+
148	Blood	Ductal shock	-	-
149	Ascites	Perforated appendicitis	*Bacillus subtilis, Streptococcus conslellatus/milleri, Bacteroides fragilis, Bacteroides thetaiotaomicron, Bacteroides uniformis*	+
150	Blood	Sepsis, Septic arthritis	-	*Staphylococcus aureus*
151	Blood	Sepsis, Septic arthritis	*Staphylococcus aureus*	*Staphylococcus aureus*
152	Synovial fluid	Sepsis, Septic arthritis	*Staphylococcus aureus*	*Staphylococcus aureus*
153	Blood	Sepsis, Septic arthritis	*Staphylococcus aureus*	*Staphylococcus aureus*
154	Blood	Sepsis, Septic arthritis	-	-
155	Blood	Sepsis, Septic arthritis	-	-

156	Blood	Sepsis, Septic arthritis	-	-
157	Blood	Sepsis, Septic arthritis	-	-
158	Blood	CGD, Pneumonitis	-	-
159	Blood	CGD, CRBSI	-	-
160	Blood	CGD enteritis	-	-
161	Blood	Sepsis	-	-
162	Blood	Sepsis	-	*Staphylococcus haemolyticus*
163	Blood	Kawasaki disease	-	-
164	Synovial fluid	Septic arthritis	-	*Streptococcus pneumoniae*
165	Synovial fluid	Septic arthritis	*Staphylococcus aureus*	*Staphylococcus aureus*
166	Pericardial effusion	Cardiac tamponade	-	-
167	Blood	Aseptic meningitis	-	-
168	Ascites	Perforated appendicitis	-	*Acinetobacter baumannii*
169	Blood	Perforated appendicitis	-	-
170	Ascites	Perforated appendicitis	-	-
171	Ascites	Perforated appendicitis	-	-
172	Blood	Inborn errors of immunity, FN	-	-
173	Blood	Parechovirus infection, Bacteremia	-	+
174	Blood	Infected liver hemangioma	-	-
175	Blood	Sepsis, Early onset GBS infection	-	*Streptococcus agalactiae*
176	Blood	UTI	-	-
177	Urine	UTI	-	*Finegoldia magna*
178	CSF	UTI	-	-
179	Blood	UTI	-	-
180	Blood	CGD enteritis	-	-
181	CSF	CGD enteritis	-	-
182	Blood	CGD enteritis	-	-
183	Blood	CGD enteritis	-	-
184	Blood	Milk allergy	-	*Staphylococcus epidermidis*
185	Blood	Meningitis, Sepsis	-	+
186	Blood	Meningitis, Sepsis	-	*Streptococcus agalactiae*

187	Blood	Meningitis, Sepsis	-	-
188	Blood	Meningitis, Sepsis	-	-
189	Blood	Meningitis, Sepsis	-	-
190	Synovial fluid	Septic arthritis	-	-
191	Blood	Enteritis	-	-
192	Abscess	Lymphadenitis	*Staphylococcus aureus*	*Staphylococcu s aureus*
193	Blood	Aspiration pneumonia	-	-
194	Blood	CRMO	-	+
195	Abscess	Lymphadenitis	-	-
196	Abscess	Lymphadenitis	*Staphylococcus aureus*	*Staphylococcu s aureus*
197	Blood	MAS, Sepsis	-	*Fusobacteriu m nucleatum*
198	CSF	Hydrocephalus	-	-
199	Blood	Dental caries, Periodontitis	-	-
200	Blood	Dental caries, Periodontitis	-	-
201	Blood	Enteritis	-	+
202	Blood	pJIA	-	-
203	Dialysis effluent	Peritonitis, Chronic renal failure	*Burkholderia cepacia*	+
204	Blood	Sepsis	-	*Streptococcus agalactiae*
205	Blood	Intracranial hemorrhage	-	-
206	Blood	Takayasu arteritis	-	-
207	Blood	Retroperitoneal abscess	-	-
208	Blood	Retroperitoneal abscess	-	-
209	Abscess	Retroperitoneal abscess	*Enterococcus faecium*	*Enterococcus faecium*
210	Blood	Sepsis, Meningitis	*Escherichia coli*	*Escherichia coli*
211	CSF	Sepsis, Meningitis	*Escherichia coli*	+
212	Blood	Necrotizing enterocolitis	-	+
213	Blood	Sepsis, Pneumonitis	*Pseudomonas aeruginosa*	*Pseudomonas aeruginosa*
214	Blood	Shock, Inborn errors of metabolism	-	-
215	CSF	Ventriculitis	-	-
216	CSF	Subdural empyema	-	-
217	Abscess	Subdural empyema	-	*Streptococcus intermedius*

218	Abscess	ALL, Brain abscess, Invasive fungal disease	-	-
219	Blood	SLE, Cardiac tamponade	-	-
220	Pericardial effusion	SLE, Cardiac tamponade	-	-
221	Pericardial effusion	SLE, Cardiac tamponade	-	-
222	Blood	Aspiration pneumonia	-	-
223	Bone tissue	CNO	-	-
224	Bone tissue	CNO	-	-
225	Blood	Sepsis	-	-
226	Blood	Sepsis	-	-
227	Blood	Brain tumor	-	-
228	CSF	Brain tumor	-	-
229	Synovial fluid	Septic arthritis	-	-
230	Blood	Bacteremia	-	*Cutibacterium acnes*
231	Blood	Sepsis	-	-
232	Blood	Neonatal asphyxia	-	-
233	Pericardial effusion	Cardiac tamponade	-	-
234	Blood	Osteomyelitis	-	-
235	Abscess	Brain abscess	*Streptococcus intermedius*	*Streptococcus intermedius*
236	Blood	Postoperative inflammation	-	-
237	Blood	Necrotizing enterocolitis	-	+
238	Blood	Ulcerative colitis	-	-
239	Blood	Ulcerative colitis	-	-
240	Blood	Pulmonary hemosiderosis	-	-
241	Blood	sJIA	-	-
242	Blood	UTI	-	-
243	CSF	UTI	-	-
244	CSF	UTI	-	-
245	CSF	UTI	-	-
246	Abscess	Tissue expander infection	*Staphylococcus aureus*	*Staphylococcus cohnii*

247	Blood	Neonatal hemochromatosis	-	-
248	Blood	Osteosarcoma, FN	-	+
249	Blood	Brain tumor, Meningitis	-	-
250	CSF	Brain tumor, Meningitis	*Enterococcus faecalis*	*Enterococcus faecalis*
251	Blood	Aspiration pneumonia	-	-
252	Blood	Aspiration pneumonia	-	-
253	Blood	Aspiration pneumonia	-	-
254	Abscess	Lymphadenitis	-	*Staphylococcu s aureus*
255	Blood	Malignant lymphoma	-	-
256	Blood	CRMO	-	*Cutibacterium acnes*
257	Blood	CRMO	-	*Cutibacterium acnes*
258	Blood	Pulmonary hemosiderosis	-	-
259	Synovial fluid	Osteomyelitis, 4p monosomy	-	-
260	Blood	Osteomyelitis	-	-
261	Abscess	Lymphadenitis	-	+
262	Abscess	Lymphadenitis	-	+
263	Abscess	Lymphadenitis	-	+
264	Blood	Kawasaki disease	-	-
265	Blood	Sepsis, Severe invasive streptococcal disease	*Streptococcus pyogenes*	*Streptococcus pyogenes*

ALL, acute lymphocytic leukemia; AML, acute myeloid leukemia; CRBSI, catheter-related bloodstream infection; CSF, cerebrospinal fluid; CGD, chronic granulomatous disease ; CRMO, chronic recurrent multifocal osteomyelitis; CVID, common variable immunodeficiency; CHD, congenital heart defects; FN, febrile neutropenia; HPS, hemophagocytic syndrome; HHV-6, human herpesvirus-6; GBS, group B streptococcus; MAS, meconium aspiration syndrome; MCTD, mixed connective tissue disorder; pJIA, polyarticular juvenile idiopathic arthritis; ; sJIA, systemic juvenile idiopathic arthritis; SLE, systemic lupus erythematosus; URI, upper respiratory infection; UTI, urinary tract infection; negative, —; positive, +.
