# Supplementary material for: Prospective Study of the Detection of Bacterial Pathogens in Pediatric Clinical Specimens Using the Melting Temperature Mapping Method
Source: Microbiol Spectr. 2022 Jun 8;10(3):e00198-22. doi: 10.1128/spectrum.00198-22 (PMC9241829; doi:10.1128/spectrum.00198-22)
Supplement: Reviewer comments [file reviewer-comments.pdf]

AMERICAN SOCIETY FOR MICROBIOLOGY | Microbiology Spectrum

Prospective study of the detection of bacterial pathogens in pediatric clinical specimens using the melting temperature mapping method

Yoji Uejima, Eisuke Suganuma, Takuma Ohnishi, Haruka Takei, Mihoko Furuichi, Satoshi Sato, Yutaka Kawano, Isao Kitajima, and HIDEKI NIIMI

Corresponding Author(s): Yoji Uejima, Saitama Children's Medical Center

Review Timeline:

Submission Date:	January 27, 2022
Editorial Decision:	February 28, 2022
Revision Received:	May 9, 2022
Accepted:	May 17, 2022

Editor: Florence Doucet-Populaire

Reviewer(s): Disclosure of reviewer identity is with reference to reviewer comments included in decision letter(s). The following individuals involved in review of your submission have agreed to reveal their identity: Kaan Çeylan (Reviewer #2); Elif Şeker (Reviewer #3)

Transaction Report:

(Note: With the exception of the correction of typographical or spelling errors that could be a source of ambiguity, letters and reports are not edited. The original formatting of letters and referee reports may not be reflected in this compilation.)

DOI: https://doi.org/10.1128/spectrum.00198-22

February 28, 2022

Dr. Yoji Uejima
Saitama Children's Medical Center
1-2 Shintoshin, Chuou-ku
Saitama 3308777
Japan

Re: Spectrum00198-22 (Retrospective study of the detection of bacterial pathogens in pediatric clinical specimens using the melting temperature mapping method)

Dear Dr. Yoji Uejima:

Thank you for submitting your manuscript to Microbiology Spectrum. When submitting the revised version of your paper, please provide (1) point-by-point responses to the issues raised by the reviewers as file type "Response to Reviewers," not in your cover letter, and (2) a PDF file that indicates the changes from the original submission (by highlighting or underlining the changes) as file type "Marked Up Manuscript - For Review Only". Please use this link to submit your revised manuscript - we strongly recommend that you submit your paper within the next 60 days or reach out to me. Detailed instructions on submitting your revised paper are below.

Link Not Available

Thank you for the privilege of reviewing your work. Below you will find instructions from the Microbiology Spectrum editorial office and comments generated during the review.

ASM policy requires that data be available to the public upon online posting of the article, so please verify all links to sequence records, if present, and make sure that each number retrieves the full record of the data. If a new accession number is not linked or a link is broken, provide production staff with the correct URL for the record. If the accession numbers for new data are not publicly accessible before the expected online posting of the article, publication of your article may be delayed; please contact the ASM production staff immediately with the expected release date.

The ASM Journals program strives for constant improvement in our submission and publication process. Please tell us how we can improve your experience by taking this quick Author Survey.

Sincerely,

Florence Doucet-Populaire

Editor, Microbiology Spectrum

Journals Department
American Society for Microbiology
1752 N St., NW
Washington, DC 20036
E-mail: spectrum@asmusa.org

Reviewer comments:

Reviewer #1 (Comments for the Author):

The present single-center study by Uejima et al., analyzes the performance the melting temperature mapping method in detection of bacteria from pediatric samples.
There are scarce information on performance the melting temperature mapping method Therefore, the study is timely and clinically interesting.
I have the following comments to make the study clearer for the reader.
Major comments

1. The standard methods should be described in detail in the method section.
2. The time interval between the test and the standard methods should be specified.
3. How did the authors define the same infectious episode?
4. What is the detailed definition of contamination pathogens in this study. Please describe.
5. The authors conclude the abstract as "Hence, the Tm mapping method could be a useful adjunct for diagnosing bacterial infections". However in describing study population it reads L113-114 "Eligible subjects, > 18 years of age, included patients suspected of bacterial infection." Very confusing. I hope that this is only a typo. Please explain.
6. What type of blood culture bottles were used in the automated blood culture system? What are the time to detection for those cultures?
7. As I understand the samples for the melting temperature mapping method and the cultures were taken simultaneously. Why is the study regarded as retrospective?
What was the blood volume in the blood culture bottles? How may bottles per patient?

Reviewer #2 (Comments for the Author):

Dear author,
Good luck for your hard work. I would like to address some of the missing points of your study, which has generally successful results. You can add these missing points to the study or specify them as limiting factors of the study in the discussion section. In addition, it would be appropriate to correct some points. These points are:
One of the most important advantages of culture in the diagnosis of bacterial infections is that antibiotic susceptibility test (AST) can be performed together with the identification process. However, as a result of the method you used in your study, AST could not be applied to the organisms, so it remains incomplete in terms of guiding the treatment.
Inoculation on the medium in bacterial infections also shows the severity of the infection, as it allows colony counting in some infections. The method used in your study is insufficient in terms of showing the bacterial load.
Even if the method used in your study could give the AST results of the infectious bacteria, since it could not distinguish between live and dead bacteria, it would also define dead bacteria as an infectious agent and cause patients to receive antibiotic treatment unnecessarily.
Even if the method used in your study was compared with the culture method, which is the gold standard test, it would have been more accurate to support the findings by using standard strains (such as ATCC) in order to give more reliable results.
Although media such as blood, chocolate, and MacConkey are used to identify bacterial infections, they often produce yeast and mold fungi that cause infection, allowing them to be identified. However, the method used in your study cannot identify yeast and mold fungi.
The LB media mentioned in line 98 should be explained.
In the material and method part, it should be explained in which media the blood culture samples, in which growth was detected by automated systems, were cultivated.
The primer used in line 149 should be explained.
In line 245, the detection of bacterial DNA by the method used in your study was interpreted as the pathogen was detected, but the expression "nucleic acid of the pathogen was detected" would be more appropriate.
In line 306, detection of dead bacteria is stated as the advantage of the method used in your study over the culture method. Explain in more detail the clinical significance of detecting dead bacteria or omit this statement in the discussion section.
Although it was stated in the introduction that the method used in your study was evaluated retrospectively, the retrospective part of the study was not clearly explained in the material and method part. The working method should be expressed more clearly in the material and method section.
Good work.

Reviewer #3 (Comments for the Author):

I have reviewed the article. I have a couple of comments to help the authors describe their work better.
1-For infections I recommend showing more references
2- With more patients, the study would be more reliable
3-Tm mapping method should be mentioned more comprehensively

Staff Comments:

Preparing Revision Guidelines
To submit your modified manuscript, log onto the eJP submission site at https://spectrum.msubmit.net/cgi-bin/main.plex. Go to Author Tasks and click the appropriate manuscript title to begin the revision process. The information that you entered when you

first submitted the paper will be displayed. Please update the information as necessary. Here are a few examples of required updates that authors must address:

• Point-by-point responses to the issues raised by the reviewers in a file named "Response to Reviewers," NOT IN YOUR COVER LETTER.
• Upload a compare copy of the manuscript (without figures) as a "Marked-Up Manuscript" file.
• Each figure must be uploaded as a separate file, and any multipanel figures must be assembled into one file.
• Manuscript: A .DOC version of the revised manuscript
• Figures: Editable, high-resolution, individual figure files are required at revision, TIFF or EPS files are preferred

For complete guidelines on revision requirements, please see the journal Submission and Review Process requirements at https://journals.asm.org/journal/Spectrum/submission-review-process. **Submissions of a paper that does not conform to Microbiology Spectrum guidelines will delay acceptance of your manuscript.** "

Please return the manuscript within 60 days; if you cannot complete the modification within this time period, please contact me. If you do not wish to modify the manuscript and prefer to submit it to another journal, please notify me of your decision immediately so that the manuscript may be formally withdrawn from consideration by Microbiology Spectrum.

If your manuscript is accepted for publication, you will be contacted separately about payment when the proofs are issued; please follow the instructions in that e-mail. Arrangements for payment must be made before your article is published. For a complete list of **Publication Fees**, including supplemental material costs, please visit ourwebsite.

Corresponding authors may join or renew ASM membership to obtain discounts on publication fees. Need to upgrade your membership level? Please contact Customer Service at Service@asmusa.org.

Thank you for submitting your paper to Microbiology Spectrum.

The present single-center study by Uejima et al., analyzes the performance the melting temperature mapping method in detection of bacteria from pediatric samples.

There are scarce information on performance the melting temperature mapping method Therefore, the study is timely and clinically interesting.

I have the following comments to make the study clearer for the reader.

Major comments

1. The standard methods should be described in detail in the method section.
2. The time interval between the test and the standard methods should be specified.
3. How did the authors define the same infectious episode?
4. What is the detailed definition of contamination pathogens in this study. Please describe.
5. The authors conclude the abstract as “Hence, the Tm mapping method could be a useful adjunct for diagnosing bacterial infections”. However in describing study population it reads L113-114 “Eligible subjects, > 18 years of age, included patients suspected of bacterial infection.” Very confusing. I hope that this is only a typo. Please explain.
6. What type of blood culture bottles were used in the automated blood culture system? What are the time to detection for those cultures?
7. As I understand the samples for the melting temperature mapping method and the cultures were taken simultaneously. Why is the study regarded as retrospective?
   What was the blood volume in the blood culture bottles? How may bottles per patient?

Christina Cuomo
Editor in Chief
Microbiology Spectrum

Dear Dr. Cuomo,

Thank you very much for your comprehensive review of our manuscript Spectrum00198-22 sent last February 28, 2022. We thank the reviewer for providing constructive comments. Here, we are sending a Word file of our revised manuscript. All the changes have been made in response to the suggestion of the reviewers.

Comment 1. The standard methods should be described in detail in the method section.

Answer 1. We have added the following detailed method to the Culture section of Materials and Methods regarding your point.
"Gram staining was performed directly from the blood culture bottle in the case of a blood culture result. In addition, aliquots of in-bottle fluid were aseptically removed from positive bottles where the bacteria had developed using standard methods and inoculated onto sheep blood agar, chocolate agar, and BTB lactose agar media. If anaerobic bacteria were presumed by Gram staining of the culture medium, the laboratory technician added isolation media for anaerobic bacteria. Blood culture bottles that did not test positive in the system for 6 days was defined as negative. For culture methods for clinical specimens other than blood, general bacterial isolation and culture intensification were performed by inoculating directly into the medium with sterile platinum ears. In addition to the common basic media, since the species of bacteria detected differed depending on the specimen, additional media including selective enrichment broths were selected by the clinical technologist based on smear results, clinical information, etc." (Pages 8, Lines 132-144)

Comment 2. The time interval between the test and the standard methods should be specified.

Answer 2. Thank you for pointing this out. A recent report said that "In less urgent situations, obtaining blood culture sets may be spaced over several hours or more" (Miller et al. Clin Infect Dis. 2018;67(6):e1-e94. doi:10.1093/cid/ciy381).

As you know, it can take time to collect more than two sets of cultures in children. We allowed a time interval of 24 h or less, as it has been reported that there is no difference in the positive rate within 24 h of a positive blood culture bottle in febrile patients (Riedel et al. J Clin Microbiol. 2008 Apr; 46(4): 1381–1385.), although the data are from adults. As you indicated, we have included the time interval in the results in the text as follows.

"The time interval of blood samples between the standard culture and the Tm mapping method was at a median of 0 h (IQR 0–0)." Pages 14, Lines 230-231.

Comment 3. How did the authors define the same infectious episode?

Answer 3. Thank you for pointing that out. The CDC/NHSN Surveillance Definitions for Specific Types of Infections 2014 states, "At the present time, NHSN does not have a set period during which only one infection of the same event type may be reported for the same patient." As you know, the definition of an episode of the same infection was unclear when we started the study in 2015. Based on the above, we also clinically evaluated each bacterial infection one case at a time.

"One bacterial infection event was clinically determined by the attending physician and the infection consulting team, one case at a time, prospectively depending on the type of infection, including signs and symptoms, laboratory results, and completion of antimicrobial therapy." Pages 7, Lines 106-109.

Comment 4. What is the detailed definition of contamination pathogens in this study. Please describe.

Answer 5. Thank you for pointing this out. We have determined nine samples of blood to be contaminating pathogens, as reported by Weinstein et al. (Clin Infect Dis. 1997;24:584-602), one case at a time, prospectively, after consultation with the attending physician and the infectious disease consulting team based on clinical symptoms and clinical course.

We have revised the following details regarding the information on Page 11-12, Lines 200-204.

"isolation of a nucleic acid of a common contaminant from the clinical specimen by the Tm mapping method without a positive result of blood culture; judged by the attending physician and Infectious Diseases Consultant to be a contaminant, and no treatment initiated, was termed "contamination pathogens"

Comment 5. The authors conclude the abstract as "Hence, the Tm mapping method could be a useful adjunct for diagnosing bacterial infections". However in describing study population it reads L113-114 "Eligible subjects, > 18 years of age, included patients suspected of bacterial infection." Very confusing. I hope that this is only a typo. Please explain.

Answer 5. We greatly appreciate your very kind remarks. As you pointed out, it is a typographical error. Our study included only cases under the age of 18. We have corrected the text. (Page 7, Line 106)

Comment 6. What type of blood culture bottles were used in the automated blood culture system? What are the time to detection for those cultures?

Answer 6. Thank you for pointing this out. We used BACT/ALERT PF Plus and FN plus (one aerobic bottle and one anaerobic bottle) blood culture bottles for the automated blood culture system used in this study. As you know, if the culture is negative, you need to wait 6-7 days. The time from filling the automated blood culture device to reporting the bacterial identification results has been added to the text as follows, and the time from DNA extraction to reporting the results of the Tm mapping method has also been included. We have added the time from filling the automated blood culture device to reporting the bacterial identification results in the text and the time from DNA extraction to reporting the results of the Tm mapping method.

"Blood was collected from subjects and aseptically incubated in BacT/ALERT® PF plus (up to 4 mL) and BacT/ALERT® FN plus (up to 10 mL) (bioMérieux, Marcy-l'Étoile, France) bottles for culture (pediatric bottles and anaerobic bottles)." （Page 7, Lines 109-112）

"If one or both bottles of a blood culture set was positive, it was counted only once as a positive blood culture set. The time for the extraction of pathogenic microorganisms is shorter if several samples collected simultaneously yield positive results." (Pages 9, Lines 125-128)

"The overall time from filling the automated blood culture device to reporting the results was as follows. The median time to report for blood samples was 6.39 days (IQR 6.15-7.01), and the median time to report for non-blood samples was 3.07 days (IQR 1.95-4.40). On the other hand, the average time from DNA extraction to reporting the results of the Tm mapping method was 3.6 hours (range 2.22-3.37)." (Page 14, Lines

237-241)

Comment 7. As I understand the samples for the melting temperature mapping method and the cultures were taken simultaneously. Why is the study regarded as retrospective?

Answer 7. The authors, especially the first author, lacked knowledge about the prospective study. In September 2014, our study on identifying pathogenic microorganisms by Tm mapping on various pediatric specimens was reviewed by our hospital's research ethics committee (No. 2014-02-013). In 2015, we began collecting specimens and performing real-time evaluations. In April 2018, we applied again to the ethics committee for funding and approval to continue the research (No. 2018-04-15).
Several specimens were collected that could be analyzed in 2020 and were analyzed and evaluated for the preparation of a paper. We misidentified these as retrospective studies; as pointed out by two reviewers, we have determined that "prospective study" is the correct term and have revised the title and text. Thank you very much for pointing out this very important point.
"Prospective study of the detection of bacterial pathogens in pediatric clinical specimens using the melting temperature mapping method" (Page 1, Line 1)
"This is a prospective single-center study" (Page 7, Line 104)

Comment 8. What was the blood volume in the blood culture bottles? How may bottles per patient?

Answer 8. Unfortunately, the amount of blood filled in each blood culture bottle was not measured; the median blood sample used for the Tm mapping method was 2 mL (IQR 1-2). Our blood culture test was performed in units of 2 bottles per set (pediatric bottle and anaerobic bottle) with a collection rate of at least 1 set of 2. The collection rate for 2 sets of 4 or more bottles was 23%.
We have added content in the respective sections as follows.
The following sentence was added to Material and Methods, " The attending physician determined the sample volume of blood culture based on clinical settings, including medical condition and weight. The attending physician collected one or two sets of blood cultures based on the patient's general condition." Page 7, Lines 112-114.
The following sentence was added to the limitation.

"Since there is no data on the amount of blood filled in the blood culture bottles, the possibility cannot be ruled out that the higher the amount of blood, the higher the positive rate of blood culture."(Page 22, Lines 383-385)

Comment 9. One of the most important advantages of culture in the diagnosis of bacterial infections is that antibiotic susceptibility test (AST) can be performed together with the identification process. However, as a result of the method you used in your study, AST could not be applied to the organisms, so it remains incomplete in terms of guiding the treatment.

Answer 9. As you point out, unfortunately, there is no antimicrobial susceptibility testing with the Tm mapping method. It remains to be determined whether the increased rate of rapid detection of pathogens with the Tm mapping method will improve patient prognosis.

If staphylococci are identified, the Tm mapping method can be used to detect the *mecA* gene, which requires an additional 45 minutes of testing. In the clinical setting, besides patient information such as the severity of illness and organs affected, the mere confirmation of GPC or GNR by Gram staining can contribute to empiric therapy. The ability to identify bacterial DNA a few hours after collecting a specimen and to confirm the presence of the bacteria in the specimen has been well received by our physicians. The authors feel that it has the potential to be very useful in clinical practice. We have added your points to the limitation along with comment 10.

Comment 10. Inoculation on the medium in bacterial infections also shows the severity of the infection, as it allows colony counting in some infections. The method used in your study is insufficient in terms of showing the bacterial load.

Answer 10. You are correct. In the culture method, the colony counts indicate the severity of the infection. This "identification test" by the Tm mapping method alone cannot indicate the severity of the infection. Although not shown in this paper, the authors have recently developed a quantification method of bacterial counts using the Tm mapping method. Our co-author is currently preparing a paper. The points raised by you are described in the limitations along with comment 9.

"In addition, this Tm mapping method does not provide information on antibiotic susceptibility tests and colony counts, which culture methods can confirm. Therefore, it

is incomplete as a guide to treatment and is not a replacement for a conventional culture test." (Pages 23, Lines 389-392)

Comment 11. Even if the method used in your study could give the AST results of the infectious bacteria, since it could not distinguish between live and dead bacteria, it would also define dead bacteria as an infectious agent and cause patients to receive antibiotic treatment unnecessarily.

Answer 11. Thank you for pointing this out. Reviewer 2 has also pointed out the issue of dead bacteria. We believe it is very important to detect dead bacteria. We are currently submitting the information shown in the figure, so please keep it confidential.
The bar graph shows the number of bacteria, and the line graph shows other biomarkers. Our research has shown that in bloodstream infections, dead bacteria in the blood are quickly scavenged. Suppose dead bacteria or DNA of dead bacteria were present in the bloodstream for a long time, as you pointed out. In that case, they will be defined as infectious bacteria and subjected to unnecessary antimicrobial therapy when detected. However, when appropriate antimicrobials are administered, dead bacteria are only detected for a limited time. The authors believe that detecting dead bacteria is not a problem but rather an advantage since they do not remain forever considering the passage of time. However, as the reviewer mentioned, I added that consideration needs to be given to the intervention methods of treatment to avoid unnecessary antimicrobial exposure.
“In addition, consideration should be given to how to intervene in treatment in light of the clinical course with respect to detection of dead bacteria to avoid unnecessary antimicrobial exposure.”（Page 23, Lines 393-395）

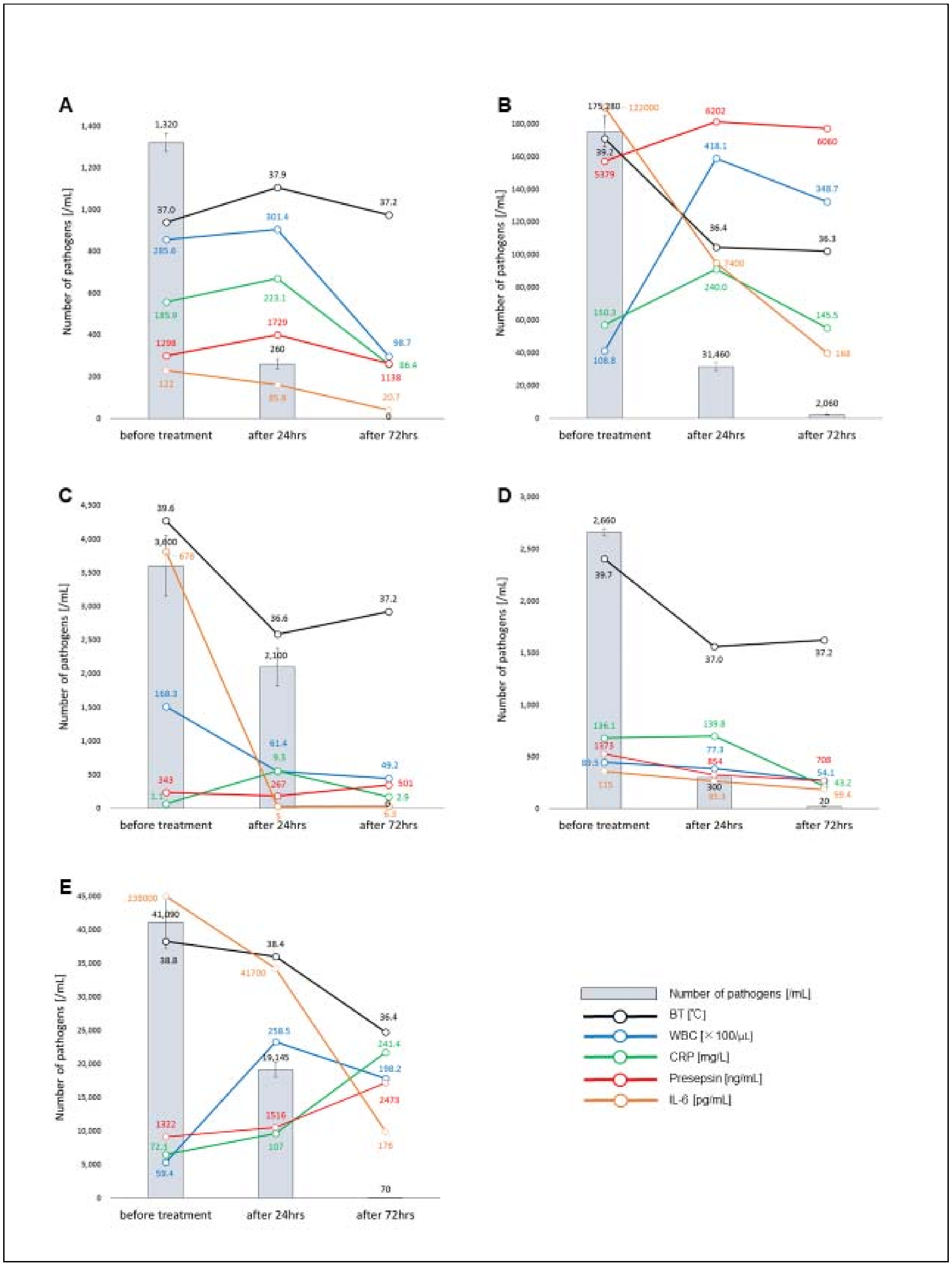

Comment 12. Even if the method used in your study was compared with the culture method, which is the gold standard test, it would have been more accurate to support the findings by using standard strains (such as ATCC) in order to give more reliable results.

Answer 12. Thank you for pointing this out. What the reviewer pointed out is true and it is an established fact that ATCC is widely used, and its quality is high. When we developed the conventional Tm mapping method, we did sensitivity testing with ATCC [Niimi et al. Sci Rep 5:12543.]
On the other hand, the JCM (Japan Collection of Microorganisms, RIKEN BioResource Research Center, Japan) Type strain was used in this study. The JCM strain used in this study has gradually been used in recent years in Japan and overseas. It has been reported in other journals in the past.
Iino T, et al. Appl Environ Microbiol. 2015;81(5):1839-1846. doi:10.1128/AEM.03741-14
Badhai J, et al. Front Microbiol. 2016 Nov 25;7:1896. doi: 10.3389/fmicb.2016.01896. PMID: 27933056; PMCID: PMC5122569.
Yoshida M, et al. EBioMedicine. 2021;64:103187. doi:10.1016/j.ebiom.2020.103187DOI:https://doi.org/10.1016/j.ebiom.2020.103187
We used JCM instead of ATCC for this experiment because of its short delivery time and ease of ordering.
To help readers understand the points you raised, we have added that the strain used in the study was the Type strain and added details about JCM. (Page 6, Lines 92-93)

Comment 13. Although media such as blood, chocolate, and MacConkey are used to identify bacterial infections, they often produce yeast and mold fungi that cause infection, allowing them to be identified. However, the method used in your study cannot identify yeast and mold fungi.

Answer 13. You are correct. The universal bacterial primer is used so that bacteria can be identified. Fungi, on the other hand, cannot be detected. Fungal universal primers and bacterial Taq are also used to search for the presence of fungi. If Candida is suspected, we perform a rapid test for 8 Candida species of fungi. [Higashi, et al. Sci Rep 10, 5828 (2020)]
We have added the following to the limitation section of the text regarding the points you pointed out,
"In the conventional culture method, fungi may be detected in the culture medium, but no fungi are detected in this method." Page 23, Lines 392-393.

Comment 14. The LB media mentioned in line 98 should be explained.

Answer 14. The following has been added.
"Luria Bertani (BD Difco^{TM}, USA) medium" (Page 6, Lines 87-88)

Comment 15. In the material and method part, it should be explained in which media the blood culture samples, in which growth was detected by automated systems, were cultivated.

Answer 15. We have provided details regarding the type of culture medium in the Culture section. (This has been included in Comment 1).

Comment 16. The primer used in line 149 should be explained.

Answer 16. Thank you for pointing this out. To make it easier for readers to understand the primers, we have added the sentence, "the bacterial conserved region of the 16S rRNA gene, which is a primer for PCR detection of all bacteria" (Page 10, Line 161-162)
Thank you for pointing this out. To make it easier for readers to understand the primers, we have added the following sentence.

Comment 17. In line 245, the detection of bacterial DNA by the method used in your study was interpreted as the pathogen was detected, but the expression "nucleic acid of the pathogen was detected" would be more appropriate.

Answer 17. Thank you very much. We have corrected the text as you indicated. (Page 12, Lines 198-201)

Comment 18. In line 306, detection of dead bacteria is stated as the advantage of the method used in your study over the culture method. Explain in more detail the clinical significance of detecting dead bacteria or omit this statement in the discussion section.

Answer 18. I would like to thank you for your very important comments regarding this "detection of dead bacteria". You also pointed this out in comment 11. We believe that

the detection of dead bacteria is controversial, as opota et al. note that "the use of amplification-based nucleic acid methods such as PCR must face several limitations associated with blood samples: 1) the presence of PCR inhibitors, 2) the presence of large amounts of non-microbial nucleic acids, 3) the presence of contaminating DNA, and 4) the presence of contaminants in the blood sample. (2) residual DNA from dead microorganisms," it states. [Opota O, et al. Clin Microbiol Infect. 2015 Apr;21(4):323-31. doi: 10.1016/j.cmi.2015.02.005.]

We recognize that this Tm mapping method is also a limitation at this time because it is PCR-based.

On the other hand, as mentioned in the main text, since the pathogenic microorganisms cannot be identified after antimicrobial administration, there is concern that broad-spectrum antimicrobials may be used or continued without de-escalation. We believe that the detection of bacterial DNA that shows signs of infection and is normally undetectable in a normally sterile area, whether viable or dead, is a significant additional piece of information for us as clinicians.

As I responded to Comment 11, we have developed a method for quantifying bacterial DNA using the Tm mapping method, and a co-author is currently working on a paper. We believe that detecting dead bacteria is a preliminary step toward developing "bacterial quantification" as a new indicator for monitoring bacterial infections and will be our future work.

We have corrected the points raised by the reviewer so that readers can understand the clinical significance of detecting dead bacteria without misunderstanding.

“Opota et al. (36) stated that one of the limitations that must be faced with respect to detecting bacteria by PCR amplification in blood samples is the presence of DNA from dead microorganisms. The Tm mapping method also uses PCR-based amplification of bacterial DNA. It is a testing method that also detects dead bacteria. It is necessary to avoid defining dead bacteria as infectious and subjecting patients to unnecessary antibiotic therapy. However, we reported a case in which nucleic acid of Streptococcus pneumoniae was detected in the cyst of a patient with an active, infected simple renal cyst after antimicrobial therapy, and although culture was negative, antimicrobial de-escalation could be performed based on the results of the Tm mapping method (37). Thus, the Tm mapping method can be very useful as a test for some of the clinical information about bacterial infections, especially in identifying the nucleic acids of dead bacteria in patients who have received prior antimicrobial therapy.” Pages 20, Lines 325-336.

Comment 19. Although it was stated in the introduction that the method used in your study was evaluated retrospectively, the retrospective part of the study was not clearly explained in the material and method part. The working method should be expressed more clearly in the material and method section.
Good work.

Answer 19. Thank you for pointing this out. It was also pointed out by Reviewer 1, so I have included it in Answer 7.

Comment 20. For infections I recommend showing more references

Answer 20. Thank you for pointing this out. I have added specific references for each infection.

Comment 21. With more patients, the study would be more reliable

Answer 21. You are correct in what reviewer 3 pointed out. Due to the outbreak of coronavirus infections, specimens were not collected as much as expected. This study was funded by a research grant, and since the study period has ended, we would like to report the data with the number of patients at this point in time.
"The present study has confounding factors and biases, such as those in the ages of pediatric patients from whom clinical specimens were collected and the small number of clinical specimens obtained." Page 22, Lines 377-379.

Comment 22. Tm mapping method should be mentioned more comprehensively

Answer 22. Thank you very much. I have added the following to the introduction regarding comprehensive references.
"This method can also prove the absence of bacteria because it uses a universal bacterial primer. On the other hand, it cannot identify multiple bacteria." Page 4, Lines 65-66.

We believe that we have addressed the reviewers' comments and hope that the revised manuscript is now acceptable for publication in *Microbiology Spectrum*. We would be glad to respond to any further questions and comments that you may have. Thank you for your generous consideration.

Sincerely,

Yoji Uejima
1-2 Shintoshin, Chuou-ku, Saitama 330-8777, Japan.
Phone: +81-48-601-2200
Fax: +81-48-601-2201
Email: uejima.yoji@saitama-pho.jp

May 17, 2022

Dr. Yoji Uejima
Saitama Children's Medical Center
1-2 Shintoshin, Chuou-ku
Saitama 3308777
Japan

Re: Spectrum00198-22R1 (Prospective study of the detection of bacterial pathogens in pediatric clinical specimens using the melting temperature mapping method)

Dear Dr. Yoji Uejima:

Your manuscript has been accepted, and I am forwarding it to the ASM Journals Department for publication. You will be notified when your proofs are ready to be viewed.

The ASM Journals program strives for constant improvement in our submission and publication process. Please tell us how we can improve your experience by taking this quick Author Survey.

As an open-access publication, Spectrum receives no financial support from paid subscriptions and depends on authors' prompt payment of publication fees as soon as their articles are accepted. You will be contacted separately about payment when the proofs are issued; please follow the instructions in that e-mail. Arrangements for payment must be made before your article is published. For a complete list of **Publication Fees**, including supplemental material costs, please visit ourwebsite.

ASM policy requires that data be available to the public upon online posting of the article, so please verify all links to sequence records, if present, and make sure that each number retrieves the full record of the data. If a new accession number is not linked or a link is broken, provide production staff with the correct URL for the record. If the accession numbers for new data are not publicly accessible before the expected online posting of the article, publication of your article may be delayed; please contact the ASM production staff immediately with the expected release date.

Corresponding authors may join or renew ASM membership to obtain discounts on publication fees. Need to upgrade your membership level? Please contact Customer Service at Service@asmusa.org.

Thank you for submitting your paper to Spectrum.

Sincerely,

Florence Doucet-Populaire
Editor, Microbiology Spectrum

Journals Department
American Society for Microbiology
1752 N St., NW
Washington, DC 20036
E-mail: spectrum@asmusa.org

Supplemental Material: Accept
